# Use of Intraperitoneal Lidocaine in Horses Undergoing Laparotomy for Colic

**DOI:** 10.3390/ani16111616

**Published:** 2026-05-26

**Authors:** Federica Giulivi, Sara Nannarone

**Affiliations:** 1 Department of Veterinary Medicine, Veterinary Teaching Hospital, University of Perugia, Via San Costanzo 4, 06126 Perugia, Italy; sara.nannarone@unipg.it; 2 Department of Veterinary Medicine, CeRiDA (Centro di Ricerca sul Dolore Animale), University of Perugia, Via San Costanzo 4, 06126 Perugia, Italy; 3 Department of Veterinary Medicine, CRCS (Centro di Ricerca sul Cavallo Sportivo), University of Perugia, Via San Costanzo 4, 06126 Perugia, Italy

**Keywords:** analgesia, colic surgery, equine, intraperitoneal lidocaine, postoperative pain

## Abstract

Perioperative lidocaine is widely used in horses with acute abdomen for its analgesic, prokinetic, anti-inflammatory, and anti-endotoxic effects, although evidence of its visceral analgesic efficacy remains inconclusive. Given the potential of alternative routes of administration for local anaesthetics, this prospective, randomised, unblinded clinical trial evaluated whether intraperitoneal (IP) lidocaine improves early postoperative recovery, including recovery from anaesthesia and the first 24 h after laparotomy for colic. A Multifactorial Numerical Rating Composite Pain Scale for equines was used for pain assessment. Covariates including aetiology, times of surgery, anaesthesia and recovery, and eventual postoperative constant rate infusion (CRI) of lidocaine were further analysed. Fifty-four horses were enrolled and equally distributed between two groups differing from the IP administration of 2 mg/kg lidocaine (group L) or not (group C) at the end of laparotomy. Postoperative lidocaine CRI (*p* = 0.007), higher ASA status (*p* = 0.005), longer recovery time (*p* = 0.0012), and large intestinal disease compared with small intestinal disease (*p* = 0.006) were significantly associated with higher pain scores, particularly within the first 24 h. No horse showed signs of toxicity. Although group L demonstrated a trend toward faster pain reduction, IP lidocaine did not significantly improve early postoperative pain outcomes. Postoperative ‘lidocaine CRI’ was associated with more severe cases, questioning its analgesic efficacy. Further research is warranted to optimise safe and effective protocols.

## Simple Summary

Lidocaine is commonly used to manage pain and support intestinal motility in horses with gastrointestinal disease undergoing laparotomy. However, its effectiveness when administered directly into the abdominal cavity is not well established. This study evaluated whether intraperitoneal lidocaine improves recovery from general anaesthesia and early postoperative pain in horses undergoing colic surgery. Fifty-four horses were randomly assigned to receive lidocaine into the abdominal cavity at the end of surgery or no treatment. Pain and recovery were monitored during the first 24 h using a standardised scoring system. Pain decreased over time in all horses. Horses receiving lidocaine showed a slight trend toward faster pain reduction, but no significant differences were observed between groups. No adverse effects were detected. These results indicate that intraperitoneal lidocaine is safe but provides limited benefit for early postoperative analgesia. The findings help clinicians make evidence-based decisions about pain management after colic surgery and may contribute to improving welfare and recovery in horses.

## 1. Introduction

The need for alternative strategies to reduce pain and discomfort in horses undergoing invasive procedures, such as laparotomy for colic, arises from the well-documented adverse effects associated with commonly used analgesics. Non-steroidal anti-inflammatory drugs (NSAIDs), although widely administered, are associated with significant side effects [1]. Similarly, conventional opioids, despite their proven efficacy in many species, present important limitations in equine practice, including decreased gastrointestinal motility, increased risk of postoperative colic, central nervous system excitation, and other complications that limit their routine use [2].

Perioperative lidocaine in horses suffering from acute abdomen has been largely used for its analgesic [3,4,5,6,7,8], prokinetic [9,10,11,12,13,14], anti-endotoxaemic [15], and anti-inflammatory [16,17,18] purposes, even though some studies have shown controversial results [19]. Visceral analgesia has been demonstrated in rats and human patients, where lidocaine inhibits spinal neuronal responses to intestinal distension, reducing pain transmission and sympathetic efferent activity [4,20], which may help preserve gastrointestinal motility and support postoperative gut function. In horses, a similar visceral analgesic effect has been suggested, with limited and inconclusive experimental evidence [6]. Furthermore, intravenous lidocaine is assumed to improve postoperative ileus by modulating multiple pathways involved in gastrointestinal dysfunction, with a reduction in sympathetic outflow and circulating catecholamines, inhibition of afferent reflex pathways that suppress intestinal motility, potential direct effects on smooth muscle activity, and modulation of inflammatory cascades [13,21]. In particular, it is proposed to reduce inflammation by inhibiting neutrophil activity, downregulating NF-κB signalling, decreasing pro-inflammatory cytokines (TNF-α, IL-2, IL-8) [16,17,18] and, during reperfusion, reducing prostaglandin E2 levels and cyclooxygenase-2 expression during reperfusion injury in the ischemic jejunum [22,23]. However, despite these theoretical mechanisms, the overall clinical and experimental evidence in horses remains variable and not consistently supportive.

Lidocaine is usually administered as a constant rate infusion (CRI) within partial intravenous anaesthesia (PIVA) protocols [6,24,25,26] and during the postoperative period for its aforementioned properties. While the effects of lidocaine on somatic pain have been scientifically proven [5], evidence regarding its efficacy for visceral pain remains partially inconclusive [6,22]. Its current use is therefore mainly supported by data from human medicine, where lidocaine has been shown to reduce opioid requirements in patients undergoing abdominal surgery, with a concurrent reduction in opioid-related side effects on intestinal motility [22,27]. Additionally, the ability of lidocaine to inhibit nociceptive fibres activated by intestinal distension has been reported in rats [20]. In horses, perioperative lidocaine CRI, administered as part of a multimodal analgesic protocol, is still considered relevant and useful for managing postoperative abdominal pain and addressing postoperative ileus (POI), as pain may contribute to worsening of the clinical condition [9,28].

Moreover, local anaesthetics (LA) possess a wide variety of routes of administration and, since the 1950s [29,30], the intraperitoneal (IP) route has been investigated and progressively implemented, showing promising results in both human and veterinary medicine. In humans, IP administration has been applied in gastric surgery [31], laparoscopic gynaecological procedures [32,33,34,35,36,37], appendectomy [38,39,40], and cholecystectomy [41,42,43,44,45] and it has been associated with improved postoperative pain, reduced analgesic and opioid requirements, and shorter hospitalisation, along with a positive safety profile. In veterinary medicine, IP LA have been investigated in dogs [46,47,48,49], cats [50,51,52], and laboratory animals [53,54,55,56], with only a pharmacokinetic study of IP lidocaine performed in horses [57]. In dogs and cats, IP LA have been associated with improved postoperative pain, particularly when included in a multimodal analgesic protocol [48,50]. In laboratory animals, additional benefits have been demonstrated, including improved survival in rats with induced faecal peritonitis, reduced oxidative stress and adhesion formation and modulation or prevention of induced chemical peritonitis [53,54,55,56].

Therefore, the aim of this study was to assess the efficacy and safety of IP lidocaine in providing postoperative analgesia in horses undergoing laparotomy for colic. This included the assessment of measurable outcomes, such as recovery scores and pain scores during the first days after laparotomy, and the absence of side effects likely attributable to lidocaine overdose. Favourable results might encourage researchers to consider this route of administration as a viable new analgesic approach in equines with acute abdomen. In line with its widespread use in human medicine, the administration of IP lidocaine could substantially improve postoperative pain in horses as well.

Furthermore, given the different scenarios that can arise from the actual causes of colic, a secondary aim of the study was to investigate all the factors that potentially influence pain scores after surgery.

## 2. Materials and Methods

### 2.1. Experimental Design

The study was approved by the Bioethical Committee of the University of Perugia, Italy (protocol n° 43782 of 13 May 2020). Written informed consent was obtained from all owners.

Client-owned horses undergoing surgery for colic at the Veterinary Teaching Hospital of the Department of Veterinary Medicine, University of Perugia, were enrolled in this prospective, randomised, unblinded clinical study. Inclusion criteria were horses older than 1 year requiring ventral midline celiotomy under general anaesthesia for gastrointestinal (GI) disease and surviving at least 24 h postoperatively. Donkeys, miniature ponies, and pregnant mares were excluded. Horses were randomised by lottery into two groups: one receiving IP lidocaine at the end of surgery (group L) and one not receiving IP lidocaine (group C).

Animals admitted for surgical resolution were assigned an ASA physical status category based on clinical evaluation. When an electronic scale was unavailable, body weight was estimated using the formula:

(Heart girth^2^ × Body length)/11877

A 14-gauge IV catheter (Terumo, Terumo Italia S.r.l., Roma, Italy) was aseptically placed in the right jugular vein. Anti-inflammatory (flunixin meglumine 1.1 mg/kg IV, Flunifen^®^, Ceva Salute Animale S.p.A., Milano, Italy) and antibiotic therapy (gentamicin 6.6 mg/kg IV and penicillin G procaine 25,000 IU/kg IM) (Aagent^®^, FATRO S.p.A, Ozzano dell’Emilia, Italy and Depocillina^®^, MSD Animal Health S.r.l, Rahway, NJ, USA) were administered. Premedication consisted of xylazine (Nerfasin^®^ 0.6 mg/kg btw IV, P.H. Farmaceutici S.r.l., Milano, Italy) and butorphanol tartrate (Dolorex^®^ 0.02 mg/kg btw IV, MSD Animal Health S.r.l, NJ, USA), followed by induction with diazepam (Ziapam^®^ 0.04 mg/kg btw IV, Ecuphar Veterinaria SLU, Barcelona, Spain) and ketamine (Ketavet^®^ 2.2 mg/kg btw IV, Intervet productions S.r.l., Aprilia, Italy) in a padded induction box. Orotracheal intubation was performed once recumbent. Additional boluses of ketamine (0.2–0.5 mg/kg) and/or thiopental sodium (0.3–0.5 mg/kg) (Pentothal sodium^®^, MSD Animal Health S.r.l., NJ, USA) could be administered at the anaesthetist’s discretion. Anaesthesia was maintained using PIVA based on isoflurane (Vetflurane^®^, Virbac, Carros, France) in 100% oxygen and a CRI of lidocaine (50 µg/kg/min) (Lidor^®^, VetViva Richter GmbH, Wels, Austria) without a loading dose [25]. Vital parameters, including cardio-respiratory values (heart rate, respiratory rate, arterial blood pressure, end-tidal carbon dioxide, arterial oxygen saturation of haemoglobin) and end-tidal isoflurane concentration, were continuously monitored and recorded every 5 min. Mean arterial blood pressure was maintained ≥70 mmHg by infusing dobutamine (Dobutamina^®^, Bioindustria L.I.M. spa, Novi Ligure, IT) to effect. Intravenous Lactated Ringer’s solution (LRS, S.A.L.F. S.p.A, Cenate Sotto, Italy) (5–10 mL/kg/h) was administered during surgery. The viscera and peritoneum were continuously rinsed with sterile LRS and approximately 5 L were left in the abdomen before closure. The lidocaine CRI was discontinued 20–30 min before the end of surgery (at closure of the abdominal fascia, endCRI).

Horses in group L received 2 mg/kg of 2% lidocaine hydrochloride IP (final dilution of approximately 0.02%). The drug was aseptically drawn into 50 mL syringes (Pikdare S.p.A., Casnate Con Bernate, Italy) and administered via a 150-centimetre extension set (B. Braun SE, Melsungen, Germany) inserted by the surgeon deeply into the abdomen through the last abdominal fascia suture before its final closure. No sedation was administered for recovery, to limit its potential influence on recovery assessment.

Recovery occurred in a padded box with assisted head and tail rope support; its quality was graded using a numeric rating scale ranging from 0 (excellent) to 4 (very poor) [58]. Times from the end of anaesthesia to extubation (extT), sternal recumbency (sterT), and standing (stanT) were recorded. The occurrence of any signs of toxicity attributable to lidocaine overdose, such as drowsiness, muscle fasciculation, convulsion, or collapse, was recorded during recovery phase and up to the time of the first postoperative pain score assessment, i.e., within 30–45 min after recovery.

During the postoperative period, all horses received the same dosages as preoperative treatment with IV flunixin meglumine twice daily for three days, followed by one daily administration for an additional two days. Antibiotic therapy consisted of IM procaine penicillin twice daily and IV gentamicin once daily for five days.

Postoperative fluid therapy was adjusted according to biochemistry results. In selected cases, at the discretion of the medical staff, a postoperative CRI of 2% lidocaine (50 µg/kg/min) was administered for 48 h, beginning with a loading dose of 1.3 mg/kg immediately after the first postoperative pain score (T1). Vital signs were recorded every 4 h. Pain was assessed using a Composite Pain Scale (CPS) at predefined time points.

The CPS (range 0–64) is a multifactorial numerical rating scale adapted from previously published composite scales for orthopaedic and visceral pain [59,60]. It integrates a comprehensive evaluation of pain by systematically considering behavioural (Behavioural Score, 14 items), social (Social Score, 4 items) and physiological parameters (Physiological Score, 11 items). The scale, following an appropriate evaluation of sensitivity, specificity, and accuracy of its parameters for acute abdomen conditions (unpublished personal data), has long been widely used in our clinic. Prior to surgery, a baseline CPS (T0) was recorded for each horse while unsedated and unrestrained in its box. The first postoperative CPS was assessed 30–45 min after recovery from anaesthesia, when horses had returned to their boxes (T1), then every 4 h during the first 24 h (T2–T7) and every 12 h on days 2 and 3 postoperatively (T8–T11). A cut-off value of 17/64 was established to distinguish absence (≤17) from presence (≥18) of pain. Pain was further classified as moderate (18–37/64) or severe (≥38/64) (Table 1). Pain assessment was performed by a single experienced operator (F.G.), with each evaluation lasting approximately 5 min per horse. Data regarding those parameters requiring longer observation time, such as defecation, urination, gastric reflux, pawing, and similar, relied also upon findings reported in the medical records.

Horses requiring rescue analgesia within the first 72 h (i.e., extra NSAIDS, alpha-2 agonists, or opioids) were not excluded from the study, but time and type of drug administration were recorded.

The following variables were recorded: breed, age, sex, weight, scheduled or emergency surgery, aetiology, presence of gastrointestinal ischemia, requirement for enterotomy and/or enterectomy, ASA status, surgery time, anaesthesia time, extT, sterT, stanT, recovery score, and requirement for postoperative lidocaine CRI (yes/no).

For horses in group L, the time between discontinuation of lidocaine CRI and IP lidocaine administration (endCRI–TIP), as well as times between TIP and extT, sterT, stanT, and T1, were recorded.

### 2.2. Statistical Analysis

Sample size was calculated a priori using repeated-measures analysis of variance to estimate the between-subject main effect of the group, as G*Power (version 3.1.9.7; Heinrich-Heine-Universität Düsseldorf, Düsseldorf, DE) does not allow direct power calculation for longitudinal ordinal outcomes. Assuming two groups, 12 repeated measurements, a significance level (α) of 0.05, 95% power, a large effect size (Cohen’s f = 0.40), a correlation among repeated measures (ρ) of 0.5, and a non-sphericity correction (ε) of 1, the required sample size was 48 horses (24 per group).

After assessment of data normality using the Shapiro–Wilk test, group comparability was evaluated using the Chi-squared (χ^2^) or Fisher’s exact test for categorical variables and the independent samples *t*-test for continuous variables. Data are reported as mean ± standard deviation or median (first–third quartiles). Pain scores were analysed using Generalised Estimation Equations (GEEs) with an autoregressive correlation structure of order 1, including group and time and their interaction. Covariates were intestinal tract involved, postoperative lidocaine CRI, intestinal compromise, enterotomy and/or enterectomy, ASA status, times for surgery, anaesthesia and recovery, and recovery score. Aetiology was analysed in a separate model to avoid multicollinearity. Linear regression was performed for individual time points and to evaluate the association between the interval from IP lidocaine administration (TIP) to T1 and the corresponding pain score. Analyses were conducted using R (version 4.5.1), with *p* ≤ 0.05 considered statistically significant.

## 3. Results

### 3.1. Study Sample

Fifty-six horses were initially included, but only fifty-four fulfilled the inclusion criteria for final enrolment in the study. Animals were divided into group L (n = 27) and group C (n = 27); two horses (one per group) were excluded due to euthanasia required within the first 24 h from recovery.

Descriptive characteristics of the groups are reported (Table 2) as well as description of the involved intestinal tract and aetiologies (Table 3). Symmetric variables are expressed as mean ± SD, while skewed variables as median with first and third quartiles (Q_1_–Q_3_) and range [min–max] when required. No statistically significant differences between groups were observed in ‘ASA status’, times of ‘anaesthesia’, ‘surgery’ and ‘recovery’, ‘quality of recovery’, lidocaine PIVA CRI total dose, and outcome (Table 2). Recovery from general anaesthesia was uneventful for all horses, and no clinical signs of toxicity were recorded in group L after IP administration.

The following breeds were represented: Warmblood (n = 24), Quarter Horse (n = 8), Thoroughbred (n = 7), Anglo-Arabian (n = 4), Arabian (n = 2) Maremmano (n = 2), Trotter (n = 2), Pure Spanish Horse (n = 1), Murgese (n = 1), Tolfetano (n = 1), Friesian (n = 1), and unknown (n = 1).

### 3.2. Pain Assessment

The number of horses that completed the pain score evaluation over the 72 h was 24/27 and 26/27 in groups L and C, respectively. In group L, twelve consecutive observations were gathered in 24/27 horses, eleven in 1/27, ten in 1/27, and nine in 1/27; in group C, twelve observations were gathered in 26/27 horses and eleven in 1/27 horses, for a total of 641 (L = 318; C = 323). Three horses in group L required one rescue analgesia each (alpha-2 agonist) at T8, T9, and T10, and shortly after rescue, they respectively were either euthanised or died spontaneously or underwent re-laparotomy. None of the horses was excluded from the statistical analysis.

The progression of pain scores assessed through time resulted in a median (Q_1_–Q_3_) value of the TS of 13 (8–18) and 12 (8–16) in groups L and C, respectively.

**Figure 1:**
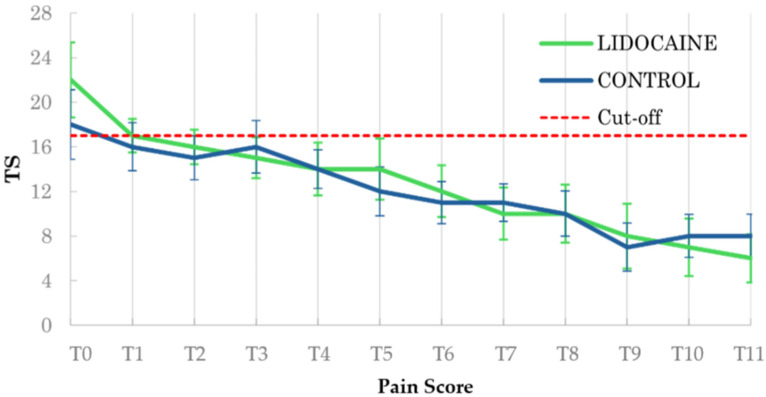
Temporal trends of the total score (TS) across study groups assessed at each CPS application. Lines denote group-specific median values and cut-off value between absence and presence of pain (TS = 17). Error bars indicate the corresponding 95% confidence intervals.

From the baseline model evaluation, no statistically significant difference between the groups in terms of score (*p* = 0.21) and pattern of pain reduction was found (*p* = 0.241); the score × time interaction was strongly significant (*p* < 0.001) (Figure 1). The intercept was 18.6 (SE = 1.59) for group L and 16.7 (SE = 1.10) for group C. The coefficients of reduction (β) for TS through time were −0.134 (SE = 0.027) and −0.102 (SE = 0.019) per hour for groups L and C, respectively. An autoregressive AR(1) correlation structure was specified, with an estimated correlation coefficient (α) of 0.82.

The advanced analysis, including clinical and temporal covariates and group-by-variable interactions, assessed baseline (intercept) pain scores of 6.87 (SE = 8.23) for group L and 5.99 (SE = 3.67) for group C. The ‘score × time’ interaction remained significant (*p* < 0.001). Postoperative ‘lidocaine CRI’ was associated with higher TS (*p* = 0.007) in both groups. Horses with ‘SI’ diseases showed lower pain scores (*p* = 0.006) than those with ‘LI’ involvement. There was a significant group L × SI interaction (*p* < 0.001) without a significant change in TS (*p* = 0.534). A higher ‘ASA status’ was also significantly associated with increased TS (*p* = 0.005). A positive and significant correlation (*p* = 0.0012) was observed between the stanT and postoperative pain scores, i.e., for each minute it took horses to stand after anaesthesia, the TS increased by an average of 0.013 points. ‘Length of surgery’ also exhibited a similar response and a trend toward significance (*p* = 0.074), with TS increasing by an estimated 0.086 (SE = 0.048) units per minute of surgery. No other statistically significant associations were found. The intra-cluster correlation (AR1) was α = 0.678. The model explains approximately 41.8% of the total variability of TS, with a 95% confidence interval ranging from 30.7% to 52.9%. This was calculated using the estimated residual variance from the GEE model (26.6 ± 2.59) and the observed total variance in TS (45.7).

From the analysis including aetiology as a covariate, the AR1 coefficient was α = 0.778 (SE = 0.041), while the estimated residual variance was 31.3 ± 3.61. This model explains 31.5% of the total variability in TS, with a 95% confidence interval ranging from 16% to 47%.

At T0, different aetiologies showed significantly different outcome levels compared with the reference category. For detailed results, please consult the Supplementary Materials (Table S1).

**Figure 2:**
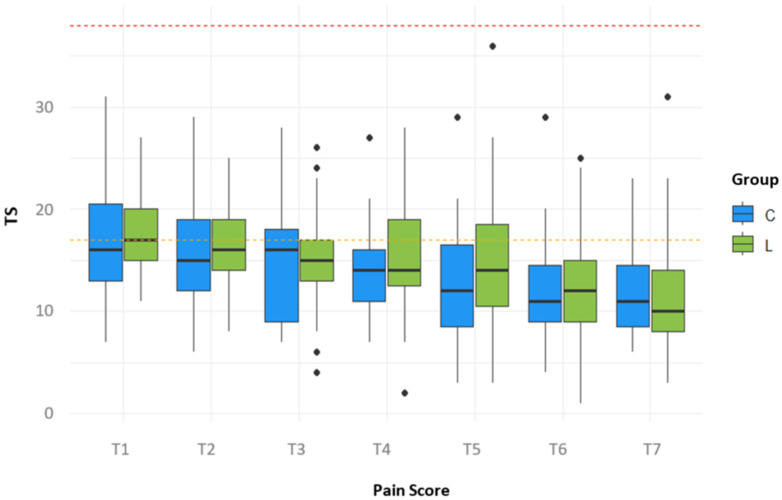
Box and whiskers plot describing results of the total score (TS) (maximum score = 64), at the first seven postoperative evaluations (T1–T7, corresponding to 0 to 24 h from recovery from anaesthesia) according to the treatment group (L = intraperitoneal lidocaine, C = control). Each box displays the median (central line) and the lower and upper quartiles (Q_1_ and Q_3_); the whiskers represent the minimum and maximum values within 1.5 times the interquartile range. The thresholds defining the limits between absence and moderate pain (orange dashed line, i.e., 17) and between moderate and severe pain (red dashed line, i.e., 38) are also indicated.

Subsequent analyses were conducted at specific time points (T1–T7) to evaluate the possible influence and extent of IP lidocaine on the analgesic effect. The statistical models accounted for the same potential covariates as before and the primary treatment effect (Figure 2).

No statistically significant association between the time elapsed from lidocaine IP and pain level registered at the first postoperative pain assessment (T1) was found (*p* = 0.939).

During the first 24 postoperative hours (T1–T7), TS was significantly associated with recovery time at T1 (*p* = 0.01) and T2 (*p* = 0.023). At T2, TS was also associated with lidocaine CRI (*p* = 0.00122), ASA status (*p* < 0.001), recovery score (*p* = 0.0183), SI disease (*p* = 0.028), enterectomy (*p* = 0.0406) and intestinal compromise (*p* = 0.0104). Significant group L interactions were observed at T2 for lidocaine CRI (*p* = 0.0201), ASA status (*p* = 0.040), recovery score (*p* = 0.00819), and SI disease (*p* = 0.001). Additional significant interactions were observed for enterotomy at T3 (*p* = 0.029) and T4 (*p* = 0.049), and for SI involvement at T3 (*p* = 0.009), T4 (*p* = 0.0113), T5 (TS *p* = 0.01, group L *p* = 0.042), T6 (TS *p* = 0.006, group L *p* = 0.0014), and T7 (TS *p* = 0.024, group L *p* = 0.032). Lidocaine CRI remained associated with TS at T6 (*p* = 0.011). All other perioperative variables were not significantly associated with TS at any time point (*p* > 0.05) (Tables S2–S8 and Figures S1–S7).

## 4. Discussion

The IP administration of 2 mg/kg lidocaine at the end of surgery resulted in a safe synergistic method for controlling pain in horses after laparotomy for colic.

When comparing pain scores of all enrolled animals, baseline TS, its temporal pattern, and the overall rate of pain reduction did not differ significantly between groups; indeed, all horses showed a marked reduction in TS over time. However, a slight difference in the reduction rate was observed in horses receiving IP lidocaine, showing a steep downward trend in TS reduction within the first 4–12 postoperative hours (i.e., up to T4) (Figure 1).

Although it is well recognised that general anaesthesia may influence pain assessment in the early postoperative period [61], potentially confounding pain scores recorded shortly after recovery, this limitation applies equally to both groups included in the present study. Therefore, any systematic effect related to residual anaesthetic or analgesic drugs is unlikely to account for differences observed between groups.

Moreover, in the study by Reed et al. [61], pain scores were reported to increase following recovery from general anaesthesia, supporting the notion that early postoperative assessments may reflect anaesthetic-related alterations rather than true nociceptive input. Consequently, the steep decline in pain scores observed in horses belonging to group L between T0 and the following four assessments in our study cannot be solely attributed to the effects of general anaesthesia or surgical resolution of the colic.

We also considered the possibility that residual effects of PIVA including lidocaine CRI could mask or overlap with the effects of its IP administration. However, as the same drug was administered via two different routes, it is more appropriate to interpret their interaction as a potential additive or combined effect rather than a masking phenomenon. Furthermore, in horses undergoing laparotomy, the elimination half-life of intraoperative lidocaine administered as PIVA in CRI has been reported to be about 65 ± 33 min [62]. Given the considerably longer time (about 130 min) between discontinuation of the intraoperative PIVA and the first postoperative pain assessment in the present study, a clinically relevant influence of lidocaine PIVA on early pain scores appears unlikely.

Since no statistically significant difference between groups suggests a possible disparity between the amount of lidocaine administered intraoperatively as PIVA, we suppose that the steep descent on the pain score may be suggestive of a timely beneficial effect of the IP administration on pain expression.

This observation aligns with findings in human gynaecological surgeries [32,33,63] and cholecystectomy [64]. In contrast, clinical studies in dogs [46,47], cats [50,52], and experimental models in rats [53,54,55] did not specifically assess early postoperative pain modulation. Similarly, in the only pharmacokinetic study conducted in horses, the half-life of intraperitoneal lidocaine was reported to be 69.51 ± 32 min when administered over a 20 min infusion [57]. As in the present study, since IP lidocaine was rapidly injected, its effective half-life may have been even shorter, suggesting that its primary benefit would occur during the immediate recovery phase, when pain scoring systems, such as our CPS, are not applicable.

Nevertheless, we consider that the beneficial effects of IP lidocaine reported in other species may extend beyond its systemic elimination half-life. In particular, its anti-inflammatory and local anaesthetic properties may contribute to sustained clinical effects that are not strictly dependent on detectable plasma concentrations, potentially influencing postoperative recovery beyond the expected pharmacokinetic profile.

In this context, evidence from experimental models supports a potential protective effect against experimentally induced peritonitis [53,55]; however, in the study by De Estrada [57], all horses receiving 30 mg/kg of IP lidocaine developed suppurative peritonitis. In contrast, no signs of peritonitis were observed in any of the horses included in the present study. The suppurative peritonitis observed in De Estrada’s study was attributed to the retention of a Foley catheter, which was aseptically placed and used for sequential sampling of peritoneal fluid. According to the author, this catheterization created a condition resembling postoperative peritonitis following colic surgery. However, we disagree with this interpretation, as none of the subjects in our study, after undergoing actual colic surgery, developed either septic or aseptic peritonitis. It is likely that the routine use of perioperative antibiotic prophylaxis may have provided additional protection against the development of peritonitis in our population.

Regarding safety, potential local cytotoxic effects of lidocaine have been reported both in in vitro and in vivo studies [65,66,67]; however, such effects appear to be primarily concentration-dependent. In the present study, no clinical evidence of peritonitis or peritoneal irritation was observed, suggesting that any local cytotoxicity was either minimal or absent under the adopted conditions. This finding may be attributable to the marked final dilution (about 0.02%) of lidocaine after IP administration, which likely contributed to reducing potential tissue damage. This is supported by the study by Karakuș et al. [67], where lidocaine cytotoxic effects were evaluated at concentrations down to 0.06%, where a dose-dependent response was observed, with minimal to negligible effects at the lowest concentration tested. In our study, the final lidocaine concentration was substantially lower than this threshold, further supporting the assumption that relevant cytotoxic effects are unlikely at such levels. However, it should be noted that in vitro colon cell models do not fully replicate the complexity of the in vivo peritoneal environment. Accordingly, further investigations using peritoneal cell populations under pathological conditions, such as colic, would be required to more accurately characterise the cytotoxic profile of lidocaine in vivo.

Finally, systemic safety considerations are also relevant. In the aforementioned study by De Estrada, one out of four horses manifested self-limiting signs compatible with acute toxicity [57]. No signs of lidocaine toxicity were observed in treated horses in our study and this is likely related to the selected low dose of 2 mg/kg, which has been established as therapeutic in animals for both antiarrhythmic and local anaesthetic purposes [68]. Furthermore, other studies in horses have reported loading intravenous doses of 2–2.5 mg/kg, followed by 50 µg/kg/min CRI, without side effects [69,70]. These data may support the safety of IP lidocaine administration after a PIVA regimen including lidocaine CRI without a prior loading dose.

When covariates were included in the model, the intercept, representing the estimated mean TS value at a specific time point after adjustment for covariates, decreased considerably, highlighting that postoperative pain expression is influenced by multiple factors and cannot be fully explained by a baseline model alone. The significant ‘score × time’ interaction confirmed that pain reduction was closely associated with temporal progression. Higher TS values were observed in horses with higher ASA status, longer surgery and recovery times, and following postoperative lidocaine CRI administration.

Higher ASA status reflects poorer clinical condition and concurrent increased anaesthetic risk [68]. Surgery duration may represent both disease complexity and surgical expertise, while prolonged recovery is frequently observed in painful or exhausted horses due to muscle damage, ATP depletion, and electrolyte imbalance [71,72,73,74]. Recovery time was positively associated with TS, supporting the relationship between prolonged recovery and immediate postoperative discomfort. In group L, the associations with ASA status and recovery time were attenuated by negative interaction terms, suggesting a partial modulation of pain expression. Recovery quality and duration are influenced by multiple factors, including surgical invasiveness, anaesthetic protocols and overall duration, body positioning, and intraoperative complications such as hypotension and intrinsic characteristics, for example, behaviour, age, sex, and body mass [75,76]. Prolonged recovery may predispose horses to complications such as neuropathy, myopathy, or hypoxaemia. Although improving pain management during this phase could theoretically enhance its quality, no significant difference was observed between groups in our study, probably due to a limited abdominal drug diffusion, or to a rather conservative lidocaine dose, or to individual variability.

Both intestinal involvement and aetiology significantly influenced the TS. Horses with SI diseases had lower TS, suggesting distinct pain expression patterns. In horses receiving IP lidocaine, the involvement of SI showed a positive, not significant interaction with the TS (i.e., it increased), possibly indicating a more complex clinical course [77].

Horses with clinical conditions associated with acute distension, ischemia, and severe inflammation presented higher TS at T0 compared to horses where distension, ischemia, or inflammation were absent. Conversely, some combinations demonstrated quicker TS decline, while others exhibited slower or potentially more complicated recovery patterns. However, many of these aetiologies were represented by a single or few subjects, limiting proper interpretation. Future studies should either exclude low-frequency aetiologies or ensure an adequate number of cases for robust statistical inference.

Lidocaine CRI is typically used for its analgesic, anti-inflammatory, and prokinetic properties [10,14,22,78] and it is commonly administered in horses with small intestinal diseases and/or with high risk of POI [22]. Therefore, its association with increased TS likely reflects its use in horses with a more severe clinical condition rather than a direct effect on pain modulation; in fact, its administration did not contribute to a clear and significant decrease in TS. However, its interaction with IP lidocaine might suggest a partial modulation of pain expression during early postoperative phases, despite marked inter-individual variability.

Over time, the influence of individual predictors decreased, and TS trajectories became more heterogeneous, indicating that the overall healing process is driven by a complex interaction of clinical and individual factors rather than single determinants.

The statistical model explained approximately 42% of TS variability, indicating moderate-to-strong explanatory capacity given the complexity of gastrointestinal disease. The remaining 58% likely reflects individual variability, prior treatments, environmental conditions, management factors, unmeasured covariates such as temperament or pain sensitivity [79], and potential measurement subjectivity, despite evaluation by a single experienced practitioner. These findings underscore the multifactorial nature of postoperative recovery after colic surgery and the need for more comprehensive data collection and objective assessment tools.

Several limitations of this study should be acknowledged. (1) The sample size was relatively small, which may limit the generalizability of the findings and reduce statistical power. (2) The CPS applied has not undergone a complete validation process; however, it has been used for years at our institution for the clinical monitoring of hospitalised animals. (3) Certain aetiologies were represented by a single horse only, precluding robust conclusions for these conditions. (4) The study was not blinded, thus introducing the potential for observer bias. (5) Additionally, the deliberately low dose of lidocaine administered, paired with its dilution, may have limited the observable therapeutic effects.

## 5. Conclusions

This study evaluated the postoperative analgesic effects and safety of IP lidocaine in horses undergoing surgery. Although no statistically significant differences were detected, a trend toward faster pain reduction in treated horses, together with the absence of observable systemic and local toxicity, suggests that IP lidocaine may provide a potential adjunctive benefit during the early postoperative phase. However, considering the known interindividual variability in lidocaine tolerance, any increase in dosage should be supported by plasma concentration monitoring to ensure safety while maintaining therapeutic efficacy.

The use of lidocaine CRI in the postoperative period was more frequently associated with clinically severe cases (SI = 70%; LI = 25%; MIX = 40%). While this finding supports its current indication in critical patients, its specific analgesic contribution remains uncertain.

Within the limits of this study, IP lidocaine appeared safe but did not demonstrate a statistically confirmed analgesic advantage. These findings highlight the need for larger, controlled, and adequately powered studies with stratified populations and objective assessment tools to determine whether IP lidocaine can provide a clinically meaningful contribution within multimodal analgesic protocols and to optimise postoperative care in horses undergoing colic surgery. 

## Figures and Tables

**Table 1:** Composite Pain Scale applied to score horses after laparotomy for colic. The scale includes 3 categories: ‘Behavioural parameters’, ‘Social parameters’, and ‘Physiological parameters’.

**Behavioural Parameters**	**Items**	**Score**
Appearance	Bright, lowered head and ears, no reluctance to move Bright and alert, occasional head movements, no reluctance to move Restlessness, pricked up ears, abnormal facial expression Excited, continuous body movements, abnormal facial expression	0 1 2 3
Position in the box	In front of the box door, observing the environment In the middle of the box, looking at the door In the middle of the box, looking at the side walls In the middle of the box, facing away from the door	0 1 2 3
Recumbency	Does not lie down or rests lying down Occasionally recumbent Frequently recumbent Constantly recumbent	0 1 2 3
Pawing at floor (number of episodes)	Quietly standing Occasional pawing (1–2 times/5 min) Frequently pawing (3–4 times/5 min) Excessively pawing (>5 times/5 min)	0 1 2 3
Flank watching	Quietly standing Occasionally (1–2/5 min) Frequently (3–4/5 min) Excessively (>4/5 min)	0 1 2 3
Kicking at abdomen	Quietly standing Occasionally (1–2/5 min) Frequently (3–4/5 min) Excessively (>4/5 min)	0 1 2 3
Sweating	No Warm or damp to touch, no sweat or wet spots visible Wet spots visible, no droplets or streams Excessive sweating, may include streams or droplets	0 1 2 3
Head posture	Above the withers Level with the withers Below the withers	0 1 2
Position and movement of the ears	Ears forward, frequent movements Ears backward or lateral and few movements	0 1
Weight shifting	No Yes	0 1
Rolling	No Occasionally (1–2 times/5 min) Frequently (3–4 times/5 min) Excessively (>5 times/5 min)	0 1 2 3
Stretching	No Yes	0 3
Depression	No Yes	0 2
Bruxism	No Yes	0 1
**Social parameters**	**Items**	**Score**
Response to opening of the stall door	Approaches the door Turns to the door Turns the other way No reaction	0 1 2 3
Response when approached	Moves closer, ears forward Look at the observer, ears forward Moves away from the observer Doesn’t move, ears are backward	0 1 2 3
Response to palpation of the peri-incisional area (postoperative only)	No reaction Mild reaction Resistance to palpation Violent reaction to palpation	0 1 2 3
Appetite	Moves towards the food and eats it readily Shows interest in hay but doesn’t eat it Neither shows interest in nor eats hay	0 1 2
**Physiological parameters**	**Items**	**Score**
Heart rate	<40 41–50 51–59 >60	0 1 2 3
Respiratory rate	<15 16–25 26–34 >35	0 1 2 3
Digestive sounds	Normal motility Decreased motility No motility Hypermotility or steel band	0 1 2 3
Rectal temperature	37–38 °C <0.5 °C variation from normality <1 °C variation from normality <2 °C or >2 °C variation from normality	0 1 2 3
Mucous membranes	Normal Abnormal	0 1
Capillary refill time (seconds)	<3 >3	0 1
Limbs extremity (warmth)	Normal Increased/decreased	0 1
Arterial pulse	Normal Increased/decreased	0 1
Defecation	Yes No	0 1
Urination	Yes No	0 1
Gastric reflux	Yes No	1 0
**TOTAL SCORE**		**__/64**

**Table 2:** Composition of the groups (L = IP lidocaine, C = control) by sex, age, body weight, emergency laparotomy, requirement for enterotomy and/or enterectomy, and presence of intestinal compromise. Data are presented as frequency, mean ± SD, or median with first and third quartiles (Q_1_–Q_3_) and range [min–max] when required. Enterotomy, enterectomy, intestinal compromise, and requirement for postoperative lidocaine CRI administration are further indicated by percentage of the total horses (n/54). * *p* < 0.05.

Descriptors	Group L	Group C
Sex distribution (n) females, geldings, stallions	12, 14, 1	13, 6, 8 * (*p* = 0.013)
Age (years)	12 ± 5	9 ± 5 * (*p* = 0.012)
Body weight (kg)	513 ± 59	509 ± 88
ASA status	4 (4–5)	4 (4–5)
Emergency laparotomy (yes, no)	25, 2	26, 1
Enterotomy (n = 37, 68% of all horses)	16	21
Enterectomy (n = 11, 20% of all horses)	6	5
Intestinal compromise (n = 34, 62% of all horses)	18	16
Time of anaesthesia (min)	134 ± 29	143 ± 42
Lidocaine PIVA total dose (mg/kg)	4.8 ± 1.6	5.2 ± 1.7
Time of surgery (min)	94.6 ± 29	106 ± 38.5
Time of recovery (min)	43 (29–57.5)	50 (38–73)
Quality of recovery	0 (0–1), range [0–3]	0 (0–1), range [0–3]
Time from TIP/endCRI and T1 (min)	130 (113–154)	158 ± 31
Postoperative lidocaine CRI (n = 22, 41% of all horses)	12 (LI = 5/15, SI = 5/9, MIX = 2/3)	10 (LI = 3/17, SI = 7/8, MIX = 0/2)
Outcome (n) discharge, death, euthanasia, re-laparotomy	20, 2, 4, 1	21, 1, 4, 1

Colic involving LI = large intestine, SI = small intestine, MIX = both large and small intestine; PIVA = partial intravenous anaesthesia, TIP = IP lidocaine administration, endCRI = end of lidocaine CRI.

**Table 3:** Incidence of aetiologies of colic and of affected tracts within the groups (L = IP lidocaine, C = control). Data are presented as frequency and percentage over the total number of subjects included in the study.

Intestinal Tract	Aetiology	Group L	Group C	%
Large intestine (LI) (n = 32, 59%)	NSS entrapment	4	3	13
	LC volvulus	1	4	9.3
	RD displacement + LC volvulus	5	0	9.3
	LC impaction	3	4	13
	RD displacement	0	2	3.6
	RD displacement + LC impaction	1	1	3.6
	GS ligament herniation	1	0	1.8
	NSS entrapment + LC volvulus	0	1	1.8
	RD displacement + LC impaction and volvulus	0	1	1.8
	LC sand impaction + caecum volvulus	0	1	1.8
Small intestine (SI) (n = 17, 32%)	Ileal impaction	2	2	7.4
	Abdominal hernia (EF entrapments L = 2, C = 1; inguinal hernia L = 1, C = 1; mesenteric hernia L = 0, C = 1; GS hernia L = 0, C = 1)	3	4	13
	Ileocecal intussusception	0	1	1.8
	Ileal hypertrophy	0	1	1.8
	Strangulating bands (omental band L = 2, C = 0; pedunculated lipoma L = 1, C = 0)	3	0	5.6
	Peritonitis + adhesions	1	0	1.8
Both (MIX) (n = 5, 9%)	RD displacement + ileal impaction	1	0	1.8
	RD displacement + ileal impaction + EF entrapment	1	0	1.8
	RD displacement + enteritis	0	1	1.8
	RD displacement + gastric impaction	0	1	1.8
	LC impaction + ileocecal intussusception + enteritis	1	0	1.8

NSS = nephrosplenic space, LC = large colon, RD = right dorsal, GS = gastro-splenic, EF = epiploic foramen, LI = large intestine, SI = small intestine, MIX = both large and small intestine.

## Data Availability

The data presented in this study are available on request from the corresponding author.

## Supplementary Materials

The following supporting information can be downloaded at: https://www.mdpi.com/article/10.3390/ani16111616/s1, Figure S1: Forest plot of the GEE model coefficients at T1 (about 30 to 45 min after recovery from general anaesthesia). The plots show the point estimates (blue dots) and 95% confidence intervals (blue horizontal bars) for each term in the model. The red dashed vertical line represents the null value (0). The terms on the y-axis include both individual and interaction terms; Figure S2: Forest plot of the GEE model coefficients at T2. The plots show the point estimates (blue dots) and 95% confidence intervals (blue horizontal bars) for each term in the model. The red dashed vertical line represents the null value (0). The terms on the y-axis include both individual and interaction terms; Figure S3: Forest plot of the GEE model coefficients at T3. The plots show the point estimates (blue dots) and 95% confidence intervals (blue horizontal bars) for each term in the model. The red dashed vertical line represents the null value (0). The terms on the y-axis include both individual and interaction term; Figure S4: Forest plot of the GEE model coefficients at T4. The plots show the point estimates (blue dots) and 95% confidence intervals (blue horizontal bars) for each term in the model. The red dashed vertical line represents the null value (0). The terms on the y-axis include both individual and interaction terms; Figure S5: Forest plot of the GEE model coefficients at T5. The plots show the point estimates (blue dots) and 95% confidence intervals (blue horizontal bars) for each term in the model. The red dashed vertical line represents the null value (0). The terms on the y-axis include both individual and interaction terms; Figure S6: Forest plot of the GEE model coefficients at T6. The plots show the point estimates (blue dots) and 95% confidence intervals (blue horizontal bars) for each term in the model. The red dashed vertical line represents the null value (0). The terms on the y-axis include both individual and interaction terms; Figure S7: Forest plot of the GEE model coefficients at T7. The plots show the point estimates (blue dots) and 95% confidence intervals (blue horizontal bars) for each term in the model. The red dashed vertical line represents the null value (0). The terms on the y-axis include both individual and interaction terms. Table S1: Results of the Generalized Estimating Equation (GEE) analysis comparing treatment groups (L vs C), with the effect of aetiology (alone) and aetiology by time interaction as covariates. The estimates (β), standard errors (SE), Wald χ^2^ values, *p*-values, and significance levels are reported; Table S2: Results of the linear regression model for TS including main effects and interaction terms between treatment groups (L vs C) and clinical, perioperative, and recovery-related variables at T1. Estimates are reported as regression coefficients (β), standard errors (SE), t-values, and *p*-values. The reference group is C. Interaction terms represent the differential effect of each predictor in group L compared to group C. NS indicates not statistically significant (*p* > 0.05); Table S3: Results of the linear regression model for TS including main effects and interaction terms between treatment group (L vs C) and clinical, perioperative, and recovery-related variables at T2. Estimates are reported as regression coefficients (β), standard errors (SE), t-values, and *p*-values. The reference group is C. Interaction terms represent the differential effect of each predictor in group L compared to group C. NS indicates not statistically significant (*p* > 0.05); Table S4: Results of the linear regression model for TS including main effects and interaction terms between treatment group (L vs C) and clinical, perioperative, and recovery-related variables at T3. Estimates are reported as regression coefficients (β), standard errors (SE), t-values, and *p*-values. The reference group is C. Interaction terms represent the differential effect of each predictor in group L compared to group C. NS indicates not statistically significant (*p* > 0.05); Table S5: Results of the linear regression model for TS including main effects and interaction terms between treatment group (L vs C) and clinical, perioperative, and recovery-related variables at T4. Estimates are reported as regression coefficients (β), standard errors (SE), t-values, and *p*-values. The reference group is C. Interaction terms represent the differential effect of each predictor in group L compared to group C. NS indicates not statistically significant (*p* > 0.05); Table S6: Results of the linear regression model for TS including main effects and interaction terms between treatment group (L vs C) and clinical, perioperative, and recovery-related variables at T5. Estimates are reported as regression coefficients (β), standard errors (SE), t-values, and *p*-values. The reference group is C. Interaction terms represent the differential effect of each predictor in group L compared to group C. NS indicates not statistically significant (*p* > 0.05); Table S7: Results of the linear regression model for TS including main effects and interaction terms between treatment group (L vs C) and clinical, perioperative, and recovery-related variables at T6. Estimates are reported as regression coefficients (β), standard errors (SE), t-values, and *p*-values. The reference group is C. Interaction terms represent the differential effect of each predictor in group L compared to group C. NS indicates not statistically significant (*p* > 0.05); Table S8: Results of the linear regression model for TS including main effects and interaction terms between treatment group (L vs C) and clinical, perioperative, and recovery-related variables at T7. Estimates are reported as regression coefficients (β), standard errors (SE), t-values, and *p*-values. The reference group is C. Interaction terms represent the differential effect of each predictor in group L compared to group C. NS indicates not statistically significant (*p* > 0.05).

## Abbreviations

The following abbreviations are used in this manuscript:

IP	Intraperitoneal
CRI	Constant Rate Infusion
ASA	American Society of Anesthesiologists
LA	Local Anaesthetic
PIVA	Partial Intravenous Anaesthesia
POI	Postoperative Ileus
GI	Gastrointestinal
IV	Intravenous
IM	Intramuscular
HR	Heart Rate
MAP	Mean Arterial Pressure
RR	Respiratory Rate
LRS	Lactate Ringer Solution
CPS	Composite Pain Scale
extT	Time to Extubation
sterT	Time to Sternal Recumbency
stanT	Time to Standing
endCRI–TIP	Interval between end of lidocaine CRI and IP lidocaine administration
α	Alpha
GEEs	Generalised Estimation Equations
Q1	First Quartile
Q3	Third Quartile
SD	Standard Deviation
TS	Total Score
β	Beta
AR(1)	Autoregressive correlation structure of order 1
LI	Large Intestine
SI	Small Intestine
MIX	Mixed Intestinal Involvement
NSS	Nephrosplenic Space
LC	Large Colon
RD	Right Dorsal
GS	Gastro-splenic Space
EF	Epiploic Foramen
NSAIDs	Non-steroidal Anti-inflammatory Drugs
