# Supplementary material for: Use of Intraperitoneal Lidocaine in Horses Undergoing Laparotomy for Colic
Source: Animals (Basel). 2026 May 26;16(11):1616. doi: 10.3390/ani16111616 (PMC13255868; doi:10.3390/ani16111616)

**LC**	Large Colon
**RD**	Right dorsal
**EF**	Epiploic foramen
**NSS**	Nephro-Splenic Space
**MIX**	Small + large intestine
**SI**	Small intestine
**NS**	Not significant

Table S1. Results of the Generalized Estimating Equation (GEE) analysis comparing treatment groups (L vs C), with the effect of aetiology (alone) and aetiology by time interaction as covariates. The estimates (β), standard errors (SE), Wald χ^2 values, p-values, and significance levels are reported.

**Predictor**	**Estimate (β)**	**Std. Error (SE)**	**Wald χ^2**	**p-value**	**Interpretation**
(Intercept)	17.52	3.31	27.92	<0.001	Positive effect on the base effect (intercept)
Time	-0.031	0.065	0.22	0.636	NS
LC impaction	-2.37	3.75	0.40	0.527	NS
LC impaction + intussusception + enteritis	10.24	3.31	9.54	0.002	Positive association with TS
Sand impaction + caecal volvulus	3.25	3.31	0.96	0.327	NS
RD displacement	-0.20	5.30	0.00	0.970	NS
RD displacement + ileal impaction	1.58	3.31	0.23	0.634	NS
RD displacement + ileal impaction + EF entrapment	-4.44	3.31	1.79	0.181	NS
RD displacement + gastric impaction	1.87	3.31	0.32	0.574	NS
RD displacement + LC impaction	3.85	5.43	0.50	0.477	NS
RD displacement + enteritis	-8.82	3.31	7.09	0.008	Negative association with TS
RD displacement + volvulus	0.58	3.95	0.02	0.884	NS
RD displacement + volvulus + LC impaction	1.32	3.31	0.16	0.690	NS
Gastrosplenic ligament herniation	2.92	3.31	0.77	0.379	NS
Internal hernia	0.35	3.81	0.01	0.927	NS
Intussusception	-6.90	3.31	4.33	0.038	Negative association with TS
Ileal hypertrophy	-0.60	3.31	0.03	0.856	NS
Strangulating lesion	-0.63	3.54	0.03	0.859	NS
NSS entrapment	-1.05	4.00	0.07	0.794	NS
NSS entrapment + volvulus	-3.47	3.31	1.09	0.295	NS
Peritonitis + adhesions	-3.28	3.31	0.98	0.323	NS
LC volvulus	3.98	4.82	0.68	0.409	NS
Time x impaction	-0.08	0.07	1.26	0.261	Interaction NS
Time x impaction + intussusception + enteritis	-0.28	0.07	18.58	<0.001	Evidence of interaction with time
Time x Sand impaction + caecal volvulus	-0.16	0.07	5.89	0.015	Evidence of interaction with time
Time x RD displacement	-0.03	0.09	0.14	0.712	Interaction NS
Time x RD displacement + ileal impaction	-0.02	0.07	0.13	0.720	Interaction NS
Time x RD displacement + ileal impaction + EF entrapment	-0.13	0.07	3.84	0.050	Evidence of interaction with time
Time x RD displacement + gastric impaction	-0.19	0.07	8.84	0.003	Evidence of interaction with time
Time x RD displacement + LC impaction	-0.17	0.09	3.44	0.064	Suggestive negative association, NS

Time x RD displacement + enteritis	0.14	0.07	4.45	0.035	Evidence of interaction with time
Time x RD displacement + volvulus	-0.08	0.07	1.36	0.244	Interaction NS
Time x RD displacement + volvulus + LC impaction	-0.19	0.07	8.50	0.004	Evidence of interaction with time
Time x Gastrosplenic ligament herniation	0.19	0.07	8.47	0.004	Evidence of interaction with time
Time x Internal hernia	-0.10	0.07	1.95	0.163	Interaction NS
Time x intussusception	-0.01	0.07	0.05	0.821	Interaction NS
Time x Ileal hypertrophy	-0.15	0.07	5.62	0.018	Evidence of interaction with time
Time x Strangulating lesion	-0.02	0.09	0.07	0.789	Interaction NS
Time x NSS entrapment	-0.10	0.07	2.21	0.137	Interaction NS
Time x NSS entrapment + volvulus	-0.08	0.07	1.66	0.197	Interaction NS
Time x Peritonitis + adhesions	-0.11	0.07	3.05	0.081	Suggestive negative association, NS
Time x LC volvulus	-0.11	0.08	1.84	0.175	Interaction NS

Table S2. Results of the linear regression model for TS including main effects and interaction terms between treatment groups (L vs C) and clinical, perioperative, and recovery-related variables at T1. Estimates are reported as regression coefficients (β), standard errors (SE), t-values, and p-values. The reference group is C. Interaction terms represent the differential effect of each predictor in group L compared to group C. NS indicates not statistically significant ($p > 0.05$).

**Predictor**	**Coefficient (β)**	**SE**	**t-value**	**p-value**	**Interpretation**
(Intercept)	16.33	9.73	1.68	0.104	NS
group L	-11.25	14.29	-0.79	0.437	Effect of group L vs reference group, NS
Postoperative lidocaine CRI (yes)	2.83	3.22	0.88	0.386	NS
MIX	-1.36	4.03	-0.34	0.737	NS
SI	-3.65	4.18	-0.87	0.390	NS
enterotomy (yes)	-1.49	3.11	-0.48	0.635	NS
intestinal compromise (yes)	1.15	3.12	0.37	0.716	NS
enterectomy (yes)	-7.69	4.26	-1.81	0.081	Suggestive negative association, NS
ASA score	0.33	2.45	0.14	0.893	NS
recovery score	-1.00	1.52	-0.66	0.513	NS
standing time	0.035	0.013	2.76	0.010	Positive association with TS
anesthesia time	-0.022	0.098	-0.22	0.826	NS
surgery time	0.022	0.110	0.21	0.837	NS
group L × enterotomy	-4.18	6.96	-0.60	0.552	Interaction NS
group L × intestinal compromise	-0.32	4.76	-0.07	0.947	Interaction NS
group L × enterectomy	0.65	8.41	0.08	0.939	Interaction NS
group L × postoperative lidocaine CRI	-4.08	4.11	-0.99	0.329	Interaction NS
group L × MIX	0.89	5.56	0.16	0.875	Interaction NS
group L × SI	2.03	6.09	0.33	0.742	Interaction NS
group L × ASA	2.54	2.96	0.86	0.398	Interaction NS

group L × recovery score	0.35	2.13	0.17	0.870	Interaction NS
group L × standing time	-0.035	0.047	-0.76	0.456	Interaction NS
group L × anesthesia time	-0.011	0.160	-0.07	0.948	Interaction NS
group L × surgery time	0.086	0.160	0.54	0.593	Interaction NS

Figure S1 - Forest plot of the GEE model coefficients at T1 (about 30 to 45 min after recovery from general anaesthesia). The plots show the point estimates (blue dots) and 95% confidence intervals (blue horizontal bars) for each term in the model. The red dashed vertical line represents the null value (0). The terms on the y-axis include both individual and interaction terms.

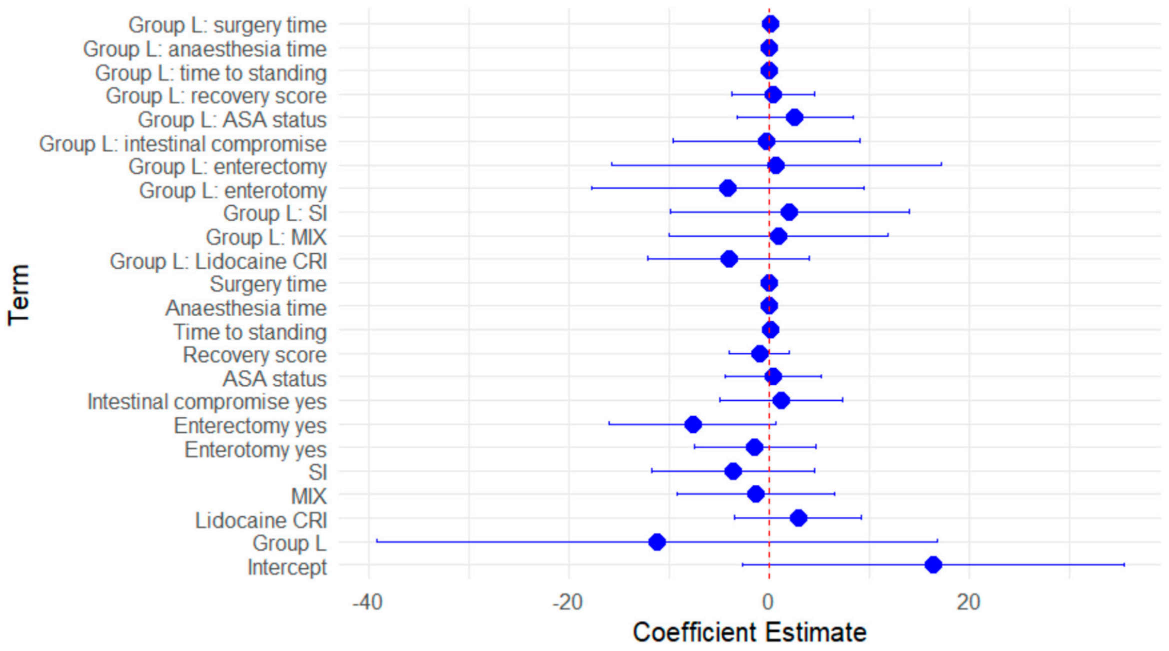

Table S3. Results of the linear regression model for TS including main effects and interaction terms between treatment group (L vs C) and clinical, perioperative, and recovery-related variables at T2. Estimates are reported as regression coefficients (β), standard errors (SE), t-values, and p-values. The reference group is C. Interaction terms represent the differential effect of each predictor in group L compared to group C. NS indicates not statistically significant ($p > 0.05$).

**Predictor**	**Coefficient (β)**	**SE**	**t-value**	**p-value**	**Interpretation**
(Intercept)	-5.336	6.692	-0.797	0.432	NS
group L	12.393	9.825	1.261	0.217	Effect of group L vs reference group, NS
Postoperative lidocaine CRI (yes)	7.901	2.213	3.571	0.00122	Positive association with TS
MIX	0.342	2.769	0.123	0.903	NS
SI	-6.643	2.874	-2.312	0.0278	Negative association with TS
enterotomy (yes)	-0.313	2.136	-0.146	0.885	NS
intestinal compromise (yes)	-5.876	2.149	-2.735	0.0104	Negative association with TS
enterectomy (yes)	-6.273	2.931	-2.141	0.0406	Negative association with TS
ASA score	6.266	1.688	3.713	0.000835	Positive association with TS
recovery score	-2.608	1.045	-2.496	0.0183	Negative association with TS
standing time	0.021	0.009	2.394	0.0231	Positive association with TS
anesthesia time	-0.049	0.067	-0.724	0.475	NS
surgery time	0.050	0.074	0.681	0.501	NS
group L × enterotomy	7.586	4.785	1.585	0.123	Interaction NS
group L × intestinal compromise	1.074	3.275	0.328	0.745	Interaction NS
group L × enterectomy	7.204	5.784	1.246	0.223	Interaction NS
group L × postoperative lidocaine CRI	-6.945	2.828	-2.455	0.0201	Evidence of interaction with group
group L × MIX	2.240	3.826	0.585	0.563	Interaction NS
group L × SI	15.158	4.189	3.618	0.00108	Evidence of interaction with group
group L × ASA	-4.377	2.039	-2.147	0.0400	Evidence of interaction with group
group L × recovery score	4.141	1.462	2.832	0.00819	Evidence of interaction with group
group L × standing time	-0.010	0.032	-0.320	0.751	Interaction NS
group L × anesthesia time	-0.154	0.113	-1.364	0.183	Interaction NS
group L × surgery time	0.181	0.109	1.657	0.108	Interaction NS

Figure S2 - Forest plot of the GEE model coefficients at T2. The plots show the point estimates (blue dots) and 95% confidence intervals (blue horizontal bars) for each term in the model. The red dashed vertical line represents the null value (0). The terms on the y-axis include both individual and interaction terms.

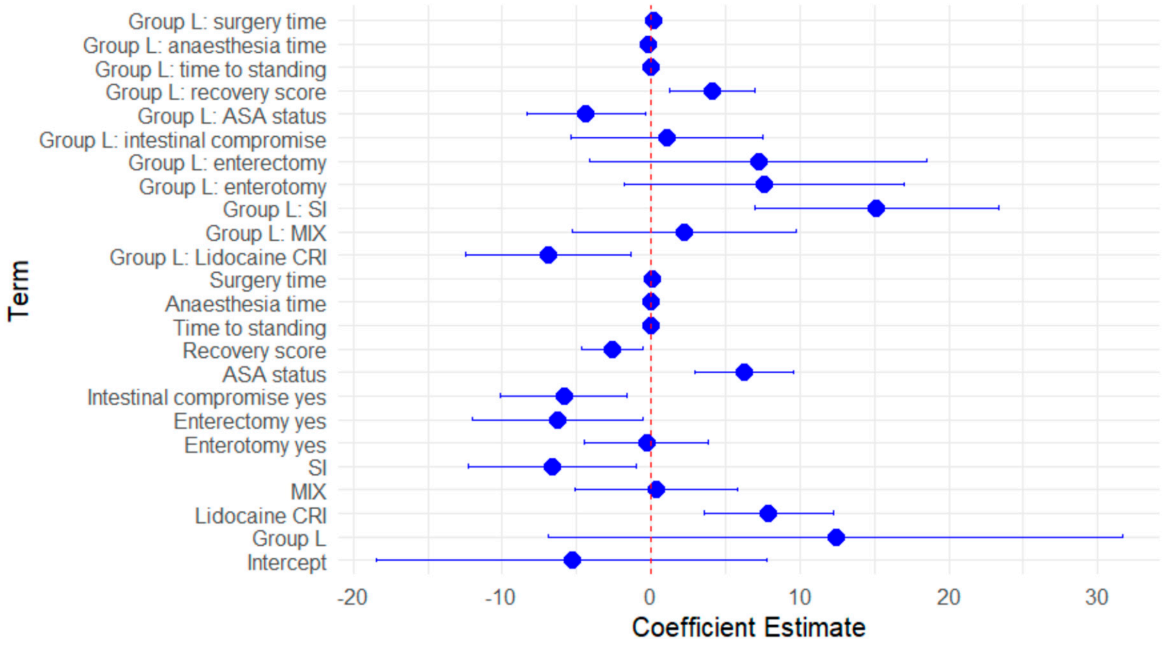

Table S4. Results of the linear regression model for TS including main effects and interaction terms between treatment group (L vs C) and clinical, perioperative, and recovery-related variables at T3. Estimates are reported as regression coefficients (β), standard errors (SE), t-values, and p-values. The reference group is C. Interaction terms represent the differential effect of each predictor in group L compared to group C. NS indicates not statistically significant ($p > 0.05$).

Predictor	Coefficient (β)	SE	t-value	p-value	Interpretation
(Intercept)	13.96	10.64	1.31	0.1995	NS
group L	-14.25	15.62	-0.91	0.3687	Effect of group L vs reference group, NS
Postoperative lidocaine CRI (yes)	4.83	3.52	1.37	0.1796	NS
MIX	-3.84	4.40	-0.87	0.3901	NS
SI	-8.96	4.57	-1.96	0.0591	Suggestive negative association, NS
enterotomy (yes)	-5.01	3.40	-1.48	0.1506	NS
intestinal compromise (yes)	-5.62	3.42	-1.65	0.1103	NS
enterectomy (yes)	-4.39	4.66	-0.94	0.3540	NS
ASA score	3.17	2.68	1.18	0.2469	NS
recovery score	-1.19	1.66	-0.72	0.4788	NS
standing time	0.02	0.01	1.76	0.0878	Suggestive positive association, not statistically significant, NS
anesthesia time	-0.17	0.11	-1.61	0.1173	NS
surgery time	0.19	0.12	1.66	0.1080	NS
group L × enterotomy	17.48	7.61	2.30	0.0287	Evidence of interaction with group
group L × intestinal compromise	0.96	5.20	0.18	0.8542	Interaction NS
group L × enterectomy	10.00	9.19	1.09	0.2854	Interaction NS
group L × postoperative lidocaine CRI	-6.46	4.50	-1.44	0.1611	Interaction NS
group L × MIX	6.44	6.08	1.06	0.2981	Interaction NS
group L × SI	18.69	6.66	2.81	0.0087	Evidence of interaction with group
group L × ASA	-1.53	3.24	-0.47	0.6396	Interaction NS
group L × recovery score	2.77	2.32	1.19	0.2430	Interaction NS
group L × standing time	0.00	0.05	0.08	0.9338	Interaction NS
group L × anesthesia time	0.09	0.18	0.51	0.6123	Interaction NS
group L × surgery time	-0.10	0.17	-0.60	0.5510	Interaction NS

Figure S3 - Forest plot of the GEE model coefficients at T3. The plots show the point estimates (blue dots) and 95% confidence intervals (blue horizontal bars) for each term in the model. The red dashed vertical line represents the null value (0). The terms on the y-axis include both individual and interaction terms.

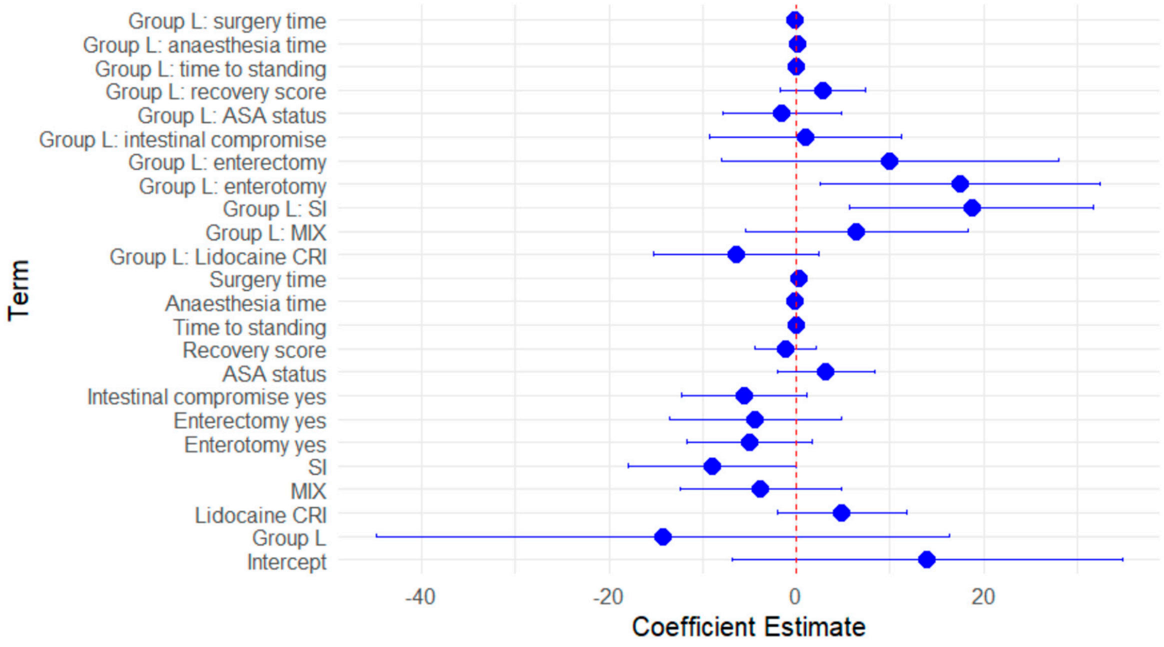

Table S5. Results of the linear regression model for TS including main effects and interaction terms between treatment group (L vs C) and clinical, perioperative, and recovery-related variables at T4. Estimates are reported as regression coefficients (β), standard errors (SE), t-values, and p-values. The reference group is C. Interaction terms represent the differential effect of each predictor in group L compared to group C. NS indicates not statistically significant ($p > 0.05$).

Predictor	Coefficient (β)	SE	t-value	p-value	Interpretation
(Intercept)	-2.48	9.92	-0.25	0.8042	NS
group L	2.98	14.57	0.21	0.8391	Effect of group L vs reference group, NS
Postoperative lidocaine CRI (yes)	4.22	3.28	1.29	0.2083	NS
MIX	-3.14	4.11	-0.77	0.4501	NS
SI	-7.81	4.26	-1.83	0.0769	Suggestive negative association, NS
enterotomy (yes)	-3.23	3.17	-1.02	0.3165	NS
intestinal compromise (yes)	-1.71	3.19	-0.54	0.5958	NS
enterectomy (yes)	-3.40	4.35	-0.78	0.4405	NS
ASA score	4.52	2.50	1.81	0.0811	Suggestive positive association, NS
recovery score	-0.79	1.55	-0.51	0.6156	NS
standing time	0.008	0.013	0.62	0.5415	NS
anesthesia time	-0.06	0.10	-0.61	0.5445	NS
surgery time	0.10	0.11	0.93	0.3593	NS
group L × enterotomy	14.56	7.10	2.05	0.0490	Evidence of interaction with group
group L × intestinal compromise	-5.34	4.86	-1.10	0.2802	Interaction NS
group L × enterectomy	6.72	8.58	0.78	0.4394	Interaction NS
group L × postoperative lidocaine CRI	-3.84	4.19	-0.92	0.3676	Interaction NS
group L × MIX	7.68	5.67	1.35	0.1859	Interaction NS
group L × SI	16.76	6.21	2.70	0.0113	Evidence of interaction with group
group L × ASA	-3.55	3.02	-1.18	0.2489	Interaction NS
group L × recovery score	1.25	2.17	0.57	0.5702	Interaction NS
group L × standing time	0.009	0.048	0.19	0.8490	Interaction NS
group L × anesthesia time	-0.07	0.17	-0.41	0.6873	Interaction NS
group L × surgery time	0.12	0.16	0.72	0.4788	Interaction NS

Figure S4 - Forest plot of the GEE model coefficients at T4. The plots show the point estimates (blue dots) and 95% confidence intervals (blue horizontal bars) for each term in the model. The red dashed vertical line represents the null value (0). The terms on the y-axis include both individual and interaction terms.

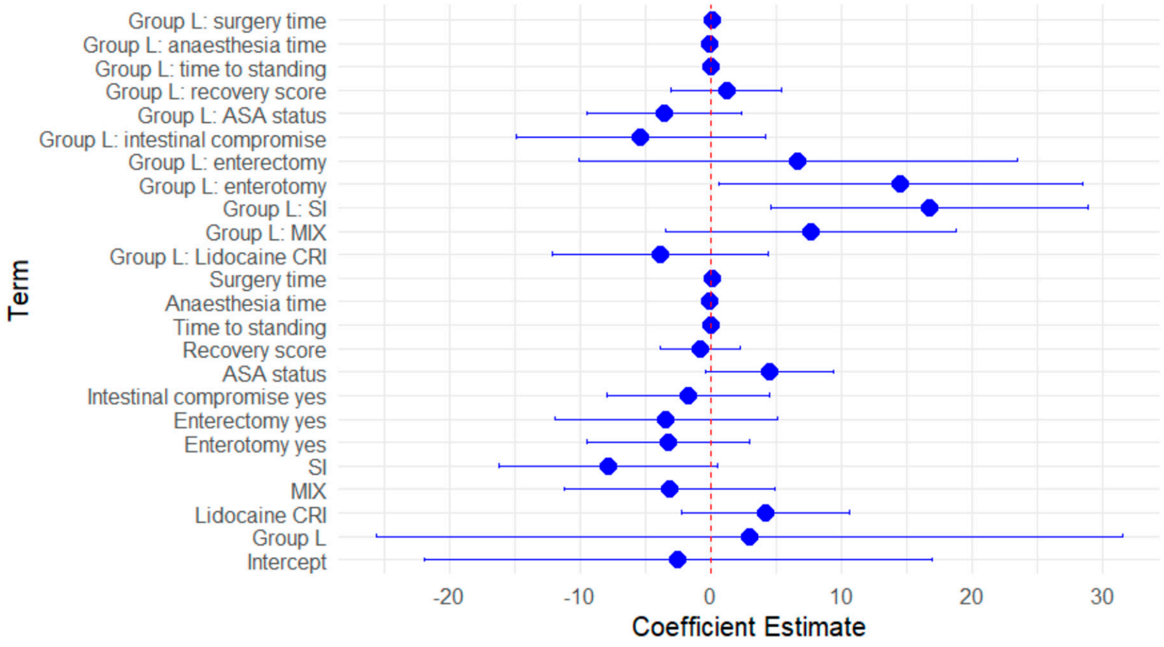

Table S6. Results of the linear regression model for TS including main effects and interaction terms between treatment group (L vs C) and clinical, perioperative, and recovery-related variables at T5. Estimates are reported as regression coefficients (β), standard errors (SE), t-values, and p-values. The reference group is C. Interaction terms represent the differential effect of each predictor in group L compared to group C. NS indicates not statistically significant (p > 0.05).

**Predictor**	**Coefficient (β)**	**SE**	**t-value**	**p-value**	**Interpretation**
(Intercept)	0.979	12.198	0.080	0.9366	NS
group L	4.693	17.909	0.262	0.7951	Effect of group L vs reference group, NS
Postoperative lidocaine CRI (yes)	7.145	4.033	1.772	0.0866	Suggestive positive association, NS
MIX	-5.818	5.046	-1.153	0.2581	NS
SI	-14.267	5.238	-2.724	0.0107	Negative association
enterotomy (yes)	-6.571	3.893	-1.688	0.1018	NS
intestinal compromise (yes)	-1.564	3.917	-0.399	0.6925	NS
enterectomy (yes)	-2.796	5.342	-0.523	0.6045	NS
ASA score	4.513	3.076	1.467	0.1528	NS
recovery score	0.668	1.904	0.351	0.7281	NS
standing time	0.002	0.016	0.107	0.9152	NS
anesthesia time	-0.091	0.123	-0.743	0.4633	NS
surgery time	0.128	0.135	0.949	0.3501	NS
group L × enterotomy	13.695	8.721	1.570	0.1268	Interaction NS
group L × intestinal compromise	-2.601	5.969	-0.436	0.6661	Interaction NS
group L × enterectomy	6.555	10.543	0.622	0.5388	Interaction NS
group L × postoperative lidocaine CRI	-4.120	5.155	-0.799	0.4304	Interaction NS
group L × MIX	5.303	6.973	0.760	0.4529	Interaction NS
group L × SI	16.255	7.636	2.129	0.0416	Evidence of interaction with group
group L × ASA	-4.048	3.716	-1.090	0.2846	Interaction NS
group L × recovery score	-1.482	2.665	-0.556	0.5822	Interaction NS
group L × standing time	-0.030	0.058	-0.506	0.6163	Interaction NS
group L × anesthesia time	0.021	0.206	0.104	0.9182	Interaction NS
group L × surgery time	0.018	0.199	0.091	0.9281	Interaction NS

Figure S5 - Forest plot of the GEE model coefficients at T5. The plots show the point estimates (blue dots) and 95% confidence intervals (blue horizontal bars) for each term in the model. The red dashed vertical line represents the null value (0). The terms on the y-axis include both individual and interaction terms.

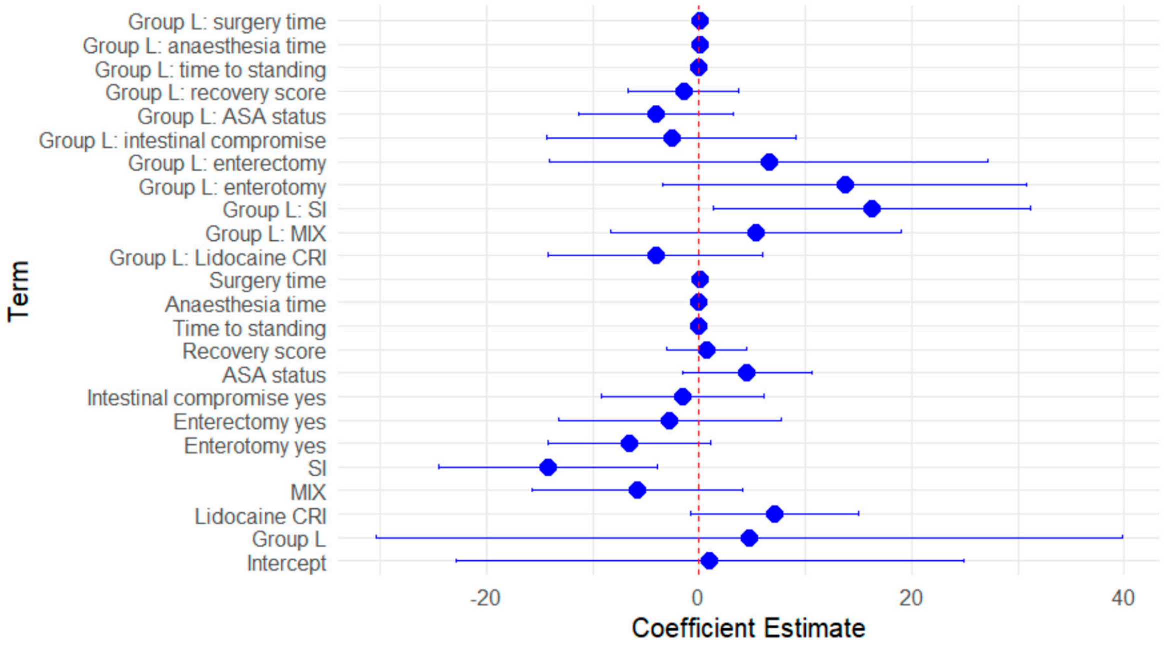

Table S7. Results of the linear regression model for TS including main effects and interaction terms between treatment group (L vs C) and clinical, perioperative, and recovery-related variables at T6. Estimates are reported as regression coefficients (β), standard errors (SE), t-values, and p-values. The reference group is C. Interaction terms represent the differential effect of each predictor in group L compared to group C. NS indicates not statistically significant ($p > 0.05$).

**Predictor**	**Coefficient (β)**	**SE**	**t-value**	**p-value**	**Interpretation**
(Intercept)	2.627602	10.125240	0.260	0.79701	NS
group L	-9.731076	14.865543	-0.655	0.51771	Effect of group L vs reference group, NS
Postoperative lidocaine CRI (yes)	9.014552	3.347629	2.693	0.01148	Positive association, NS
MIX	-1.298220	4.188884	-0.310	0.75876	NS
SI	-12.801654	4.347727	-2.944	0.00620	Negative association
enterotomy (yes)	-3.157678	3.231701	-0.977	0.33634	NS
intestinal compromise (yes)	-3.538390	3.251052	-1.088	0.28509	NS
enterectomy (yes)	-2.652511	4.434253	-0.598	0.55421	NS
ASA score	3.418096	2.553514	1.339	0.19076	NS
recovery score	-1.616409	1.580471	-1.023	0.31461	NS
standing time	0.019470	0.013226	1.472	0.15141	NS
anesthesia time	-0.075340	0.101812	-0.740	0.46506	NS
surgery time	0.100276	0.111662	0.898	0.37632	NS
group L × enterotomy	10.951083	7.239224	1.513	0.14081	Interaction NS
group L × intestinal compromise	0.764400	4.954610	0.154	0.87842	Interaction NS
group L × enterectomy	2.896007	8.751160	0.331	0.74300	Interaction NS
group L × postoperative lidocaine CRI	-7.414071	4.279324	-1.733	0.09345	Suggestive interaction with group, NS
group L × MIX	2.074579	5.788370	0.358	0.72255	Interaction NS
group L × SI	22.158663	6.338395	3.496	0.00149	Evidence of interaction with group
group L × ASA	-1.292039	3.084423	-0.419	0.67828	Interaction NS
group L × recovery score	2.737874	2.212549	1.237	0.22553	Interaction NS
group L × standing time	0.006496	0.048474	0.134	0.89429	Interaction NS
group L × anesthesia time	0.036540	0.170802	0.214	0.83205	Interaction NS
group L × surgery time	-0.025355	0.165148	-0.154	0.87901	Interaction NS

Figure S6 - Forest plot of the GEE model coefficients at T6. The plots show the point estimates (blue dots) and 95% confidence intervals (blue horizontal bars) for each term in the model. The red dashed vertical line represents the null value (0). The terms on the y-axis include both individual and interaction terms.

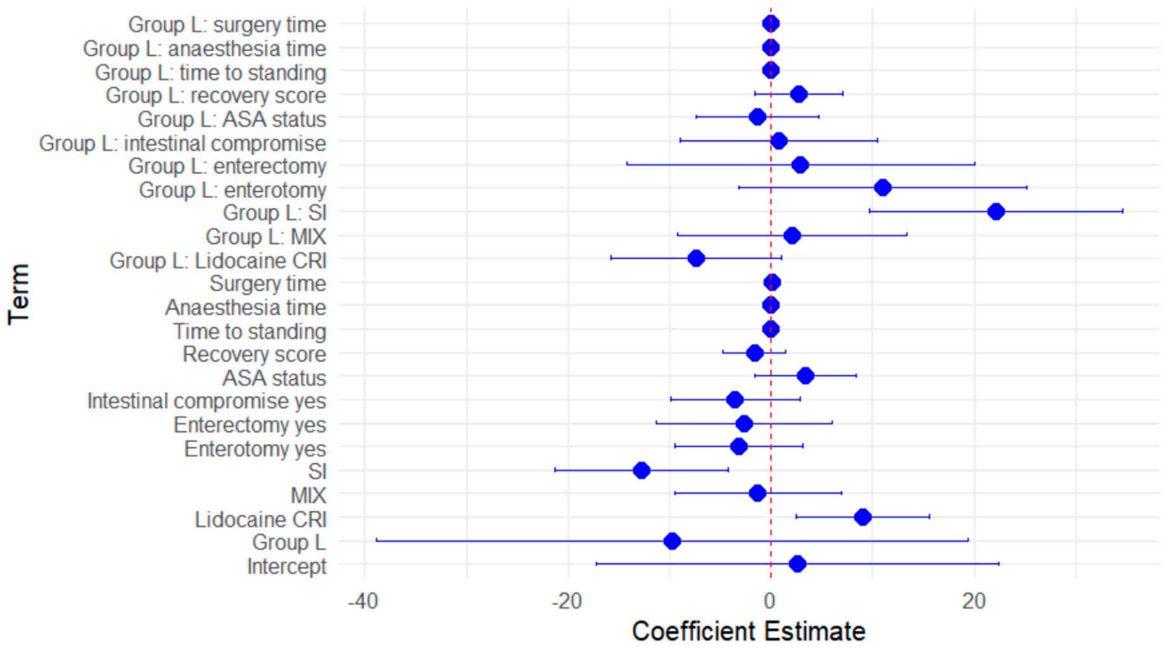

Table S8. Results of the linear regression model for TS including main effects and interaction terms between treatment group (L vs C) and clinical, perioperative, and recovery-related variables at T7. Estimates are reported as regression coefficients (β), standard errors (SE), t-values, and p-values. The reference group is C. Interaction terms represent the differential effect of each predictor in group L compared to group C. NS indicates not statistically significant ($p > 0.05$).

**Predictor**	**Coefficient (β)**	**SE**	**t-value**	**p-value**	**Interpretation**
(Intercept)	-2.256	11.070	-0.204	0.8399	NS
group L	-1.709	16.253	-0.105	0.9169	Effect of group L vs reference group, NS
Postoperative lidocaine CRI (yes)	5.801	3.660	1.585	0.1235	NS
MIX	1.833	4.580	0.400	0.6918	NS
SI	-11.259	4.754	-2.369	0.0245	Negative association
enterotomy (yes)	-3.756	3.533	-1.063	0.2963	NS
intestinal compromise (yes)	-3.527	3.554	-0.992	0.3289	NS
enterectomy (yes)	0.629	4.848	0.130	0.8977	NS
ASA score	4.562	2.792	1.634	0.1127	NS
recovery score	-1.511	1.728	-0.874	0.3889	NS
standing time	0.0015	0.0145	0.101	0.9206	NS
anesthesia time	-0.029	0.1113	-0.260	0.7963	NS
surgery time	0.053	0.1221	0.436	0.6663	NS
group L × enterotomy	8.417	7.915	1.063	0.2961	Interaction NS
group L × intestinal compromise	4.421	5.417	0.816	0.4208	Interaction NS
group L × enterectomy	0.019	9.568	0.002	0.9984	Interaction NS
group L × postoperative lidocaine CRI	-3.360	4.679	-0.718	0.4783	Interaction NS
group L × MIX	-4.974	6.329	-0.786	0.4381	Interaction NS
group L × SI	15.548	6.930	2.244	0.0324	Evidence of interaction with group
group L × ASA	-4.027	3.372	-1.194	0.2418	Interaction NS
group L × recovery score	0.759	2.419	0.314	0.7559	Interaction NS
group L × standing time	0.043	0.053	0.806	0.4265	Interaction NS
group L × anesthesia time	0.094	0.187	0.504	0.6176	Interaction NS
group L × surgery time	-0.079	0.181	-0.435	0.6664	Interaction NS

Figure S7 - Forest plot of the GEE model coefficients at T7. The plots show the point estimates (blue dots) and 95% confidence intervals (blue horizontal bars) for each term in the model. The red dashed vertical line represents the null value (0). The terms on the y-axis include both individual and interaction terms.

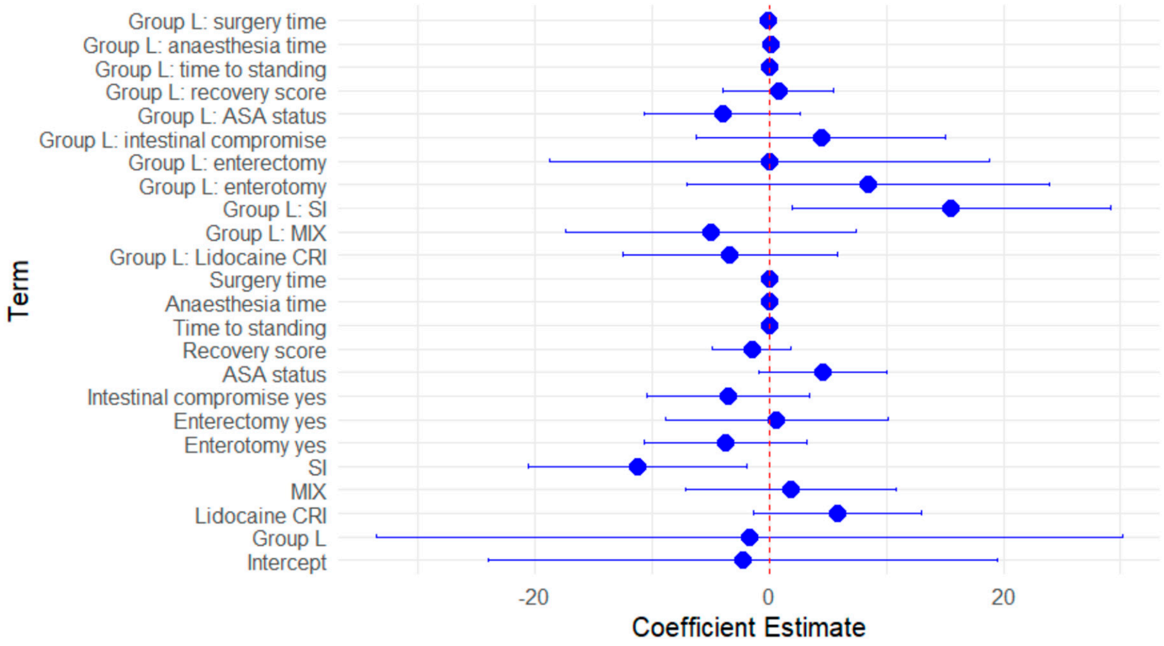

Supplement: Supplementary file 1 [file animals-16-01616-s001.zip › animals-4254696-supplementary.pdf]
